# Correlation Between the Indentation Properties and Microstructure of Dissimilar Capacitor Discharge Welded WC-Co/High-Speed Steel Joints

**DOI:** 10.3390/ma13112657

**Published:** 2020-06-11

**Authors:** Giovanni Maizza, Renato Pero, Frediano De Marco, Takahito Ohmura

**Affiliations:** 1 Department of Applied Science and Technology, Politecnico di Torino, 10129 Torino, Italy; 2 Department of Industrial Engineering, University of Rome “Tor Vergata”, 00133 Rome, Italy; renato.pero@alumni.uniroma2.eu; 3 National Interuniversity Consortium of Materials Science and Technology (INSTM), 50121 Florence, Italy; frediano.demarco@polito.it; 4 High Strength Materials Group, National Institute for Materials Science (NIMS), 1-2-1 Sengen, Tsukuba 305-0047, Ibaraki, Japan; ohmura.takahito@nims.go.jp

**Keywords:** nano-instrumented indentation test, high-speed steel, dissimilar alloy welding, steel-tungsten carbide welding, capacitor discharge welding, transitional welding layer

## Abstract

The welding of cemented carbide to tool steel is a challenging task, of scientific and industrial relevance, as it combines the high level of hardness of cemented carbide with the high level of fracture toughness of steel, while reducing the shaping cost and extending the application versatility, as its tribological, toughness, thermal and chemical properties can be optimally harmonised. The already existing joining technologies often impart either insufficient toughness or poor high-temperature strength to a joint to withstand the ever-increasing severe service condition demands. In this paper, a novel capacitor discharge welding (CDW) process is investigated for the case of a butt-joint between a tungsten carbide-cobalt (WC-Co) composite rod and an AISI M35 high-speed steel (HSS) rod. The latter was shaped with a conical-ended projection to promote a high current concentration and heat at the welding zone. CDW functions by combining a direct current (DC) electric current pulse and external uniaxial pressure after a preloading step, in which only uniaxial pressure is applied. The relatively high heating and cooling rates promote a thin layer of a characteristic ultrafine microstructure that combines high strength and toughness. Morphological analysis showed that the CDW process: (a) forms a sound and net shaped joint, (b) preserves the sub-micrometric grain structure of the original WC-Co composite base materials, via a transitional layer, (c) refines the microstructure of the original martensite of the HSS base material, and (d) results in an improved corrosion resistance across a 1-mm thick layer near the weld interface on the steel side. A nano-indentation test survey determined: (e) no hardness deterioration on the HSS side of the weld zone, although (f) a slight decrease in hardness was observed across the transitional layer on the composite side. Furthermore, (g) an indication of toughness of the joint was perceived as the size of the crack induced by processing the residual stress after sample preparation was unaltered.

## 1. Introduction

Tungsten carbide (WC) bonded with 6 to 20 wt% of cobalt (WC-Co composite, cemented carbide or hard metal) is part of a family of materials which are composed of micrometric (or sub-micrometric) WC particles embedded in a cobalt matrix. The WC particles are randomly oriented to grant isotropic properties to the composite [1]. By varying the cobalt content, a wide range of wear resistance, hardness, strength, fracture toughness, and temperature and chemical stability can be achieved. 

WC-Co composites are widely employed in harsh conditions as tools in various forming manufacturing, mining, and oil and gas applications. Most applications require excellent combinations of hardness and toughness, which cannot be achieved by a WC-Co composite alone. The joining of tool steels with WC-Co composites can provide an alternative manufacturing route which combines the toughness of tool steel with the hardness of WC-Co composites. High-speed steel (HSS) usually contains W and V elements, which impart high tribological properties at high temperatures and high fracture toughness. 

An initial attempt of industrial relevance towards the development of such an alternate manufacturing route was patented by Karl and Hans in 1935 [2]. Subsequently, other joining techniques that relied on powder metallurgy, surface processing and welding technologies were proposed. In the powder metallurgy approach, WC-Co powder may be co-sintered with stainless steel (SUS303), the latter being in the form of powder or bulk material [3], and consolidated together with tool steel (1.2419) under a high voltage electric discharge [4], sinter-welded, by means of capacitor discharging, to high-speed steel (AISI M2) [5], uniaxially hot-pressed to W alloy [6], sintered, by means of a microwave, with Co powder [7], co-sintered with steel powder [8], sintered, by means of pulsed current sintering, with stainless steel powder (SUS316L) [9] or liquid-phase sintered with an Fe-Ni alloy (Invar) [10].

The surface technologies include the cold spray deposition onto SUS 304 stainless steel [11] and carbon steel [12], vacuum plasma spray deposition (onto steel) [13] and high velocity oxygen fuel thermal spraying onto stainless steel [14] and onto AISI 4043 [15], electrophoretic deposition onto high-speed steel [16] and detonation gun spray coating on AISI 4043 [15]. 

The bulk joining approach ranges from low-temperature brazing to fusion welding by laser, laser-tungsten inert gas (TIG), TIG, electron beam, metal-arc inert gas (MIG), friction, diffusion bonding and others [17]. Specifically, WC-Co is brazed onto 410 stainless steel using Ni and Cu-Zn fillers [18], laser welded onto AISI 6542 [19] as well as onto carbon steel without any interlayer [20,21] and with an Invar interlayer [22], or TIG welded onto carbon steel using an Ni-Fe-C filler [23] or diffusion bonded to mild steel [24], or to carbon steel [25], tool steel [24] and stainless steel using an Ni interlayer [26] and with 90MnCrV8 steel using Ni-Cu alloys as a filler [27], or friction welded with JIS SKD11 tool steel [28], friction stir welded with S45C steel [29], liquid phase bonded (partial transient) to carbon steel using Ti/Ni/Ti interlayers [30], and rotary friction welded with AISI 304L [31].

However, each of these techniques may give rise to manufacturing defects, which are generally accumulated at the weld zone, such as pores, cracks, delamination, un-joined regions, embrittling second phases, residual stresses, and even spalling [8,21,24,32]. These defects can be prevented or limited by optimising the process parameters [5]. Fusion welding is relatively fast, easy to perform and more convenient (due to the lower cycle temperature) than solid-state bonding. However, the latter benefits from a minimising of the grain growth, precipitating growth and phase segregations [5].

The research results attained so far show that there does not yet exist a viable and general net-shape welding technology of WC-Co/steel joint that is able to fulfil most engineering applications. 

In this study, a capacitor discharge welding (CDW) method is proposed as an eco-friendly net-shape welding technology to produce sound and tough joints between AISI M35 and WC-10Co (10 wt% Co) composite rods. The indentation properties of the joint are investigated by means of a nano-instrumented indentation test (nIIT). The viability of the proposed technology for industrial applications is proved through a detailed investigation aimed at establishing a local cross-link between the relevant microstructure features, the chemical composition, and the indentation properties over the weld region. 

## 2. Capacitor Discharge Welding (CDW)

Capacitor discharge welding (CDW) employs a combination of a high intensity electric current pulse along with mechanical pressure [33,34,35]. The process exploits strong non-equilibrium phenomena to promote the welding between either similar or dissimilar alloys, especially difficult-to-weld materials, in order to make metal-metal or metal-ceramic (e.g., intermetallic alloys, zirconia, alumina, SiAlON and silicon nitride) joints [34,36,37,38]. The rods are secured with suitable clamps, which also act as electrodes. As the contact region between the two pressed parts opposes a higher electric resistance than the two bulk materials, a rapid preferential heating is concentrated in a narrow region near the contact interface. The electric energy previously stored in a capacitor bank is converted into a high energy density pulse. A typical current pulse is characterised by a 200–500 kA current peak and a time of 10–30 ms. The rapid heating (of the order of 10^4^ °C/s) is followed by an extremely fast conduction cooling (10^3^ °C/s). The actual heating and cooling rates in the welding region depend on the geometry and thermal conductivity of the joint as well as on the mass of the electric conduction tooling of the CDW machine [35]. In order to control the delivered energy density at the contact region, either one or two ends of the joining parts may be shaped with a spherical or conical projection [35]. The electric and thermal contact resistance exerted by the imposed mechanical pressure plays a major role in the CDW process. The welding temperature peak is generally close to the melting point. The high intensity magnetic fields, originating from the electric discharge, cause a radial outward ejection of liquid droplets from the contact region, thereby ensuring an almost solid-state joint region [33]. As a result, various metallurgical phenomena concur in the development of a unique weld microstructure. The very short pulse time makes the diffusion of any alloying elements practically negligible, although very high temperature peaks are observed at the joint interface of a CDW joint. In the same manner, as solidification may occur in a very narrow region near the interface region, due to localised incipient melting, element segregation is less likely. Moreover, the severe plastic deformation effected by the imposed mechanical pressure may promote a recrystallisation process in the solid region of the interface. 

The heat affected zone (HAZ) is usually very small, without any grain growth, due to the brevity of the heating time [33]. The interplay between the CDW-induced metallurgical phenomena, associated with the resulting fine microstructure, determines a unique combination of high strength and toughness of the CDW joint. A second current pulse may also be released, under a low imposed pressure, to relax any possible residual stresses that may have developed during the heating and cooling cycle. Alternatively, a proper post- stress relaxation heat treatment can be performed off-line using a controlled atmosphere furnace. 

## 3. Materials, Methods and Results

**Figure 1:**
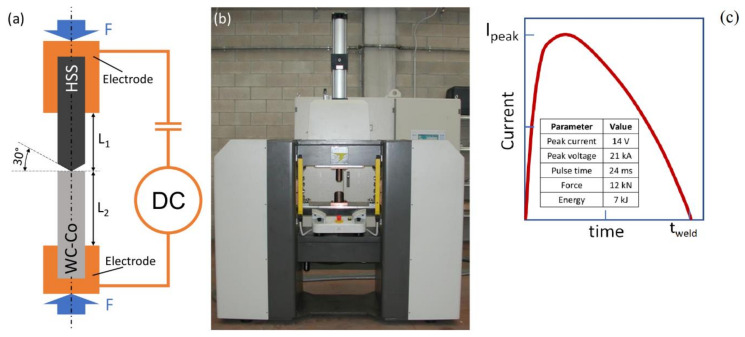
(**a**) Sketch (out of scale) of the capacitor discharge welding (CDW) tool-sample system arrangement, *F* is the pre-load set in the calibration stage, (**b**) CDW machine and (**c**) current waveform adapted from the low-resolution machine display plot.

The materials that had to be joined were supplied by Silmax S.p.A. (Lanzo, Italy) and consisted of a full density WC-Co composite rod and a drawn and tempered AISI M35 high-speed steel (HSS) rod. Both rods were 40 mm long and had a 5 mm diameter. A conical projection was machined at one end of the steel rod with an apex angle of 30° (Figure 1a). The design of the HSS microstructure after the tempering process is of paramount importance to ensure an optimal balance between the tribological and fracture toughness properties. The primary carbides are mostly spherical in shape and are up to a few micrometres in size [39,40,41], partially dissolved, spheroidised and eventually may reform. Their complex structure comprises MC (M, typically vanadium), M_6_C (M, typically tungsten) and M_23_C_6_ (M, typically chromium) types [40,42]. Mo and Fe can replace W and V in the carbides [43], with several combinations. A martensitic (or bainitic [41]) structure may also embed some residual austenite.

The designed butt joint was realised by means of capacitor discharge welding (Focusweld S.r.l., Orbassano, Italy; Figure 1) in a single shot. The two rods were firmly clamped between two hollow copper clamps. The two free parts of the rods in relative contact were 3.1 mm (L_1_) and 3.8 mm (L_2_) in length for the HSS and the composite, respectively. The first step of the CDW process is the so-called pre-loading or calibration stage. The purpose of pre-loading is to ensure that the user-set load and electric input energy are adequate for the selected materials. The imposed load was controlled by means of an on-board load cell, the displacement exerted by the welding materials was monitored by a linear variable displacement transducer (LVDT) sensor, while the electric parameters (current energy and voltage) were monitored by an on-board Rogowski current probe and a voltmeter. The calibration stage ensured that the user-setting of the load and current energy were consistent with the nature of the selected welded materials. If the user-setting (load and/or current energy) were too excessive for a selected material joint, the plastic displacement of the welding materials would also be significant, and the internal proportional-integral-derivative (PID) control system would therefore operate an automatic rescaling of the selected parameters in order to permit a safe welding. A qualitative measure of the goodness of the CDW was given by the number of ejected droplets observed after welding. A low number of liquid droplets indicated an insufficient input energy. Conversely, too many droplets indicated an excessive input energy. The user pre-set values of input direct current (DC) electric energy and pre-load before process calibration, were 5 kJ and 11 kN, respectively. After calibration, the final welding parameters (automatically set by the machine) were recorded on an internal CPU and visualized on the on-board display. These welding data were 14 V, 21 kA, 12 kN and 24 ms for the peak voltage, peak current, welding force and pulse time, respectively. In this case, due to the high hardness of the joining materials, the PID control system determined a slightly larger input energy and welding load, namely 7 kJ (= 14 V x 21 kA x 24 ms) vs. 5 kJ (pre-set) and 12 kN vs. 11 kN (pre-set). The current waveform adapted from the low-resolution machine display plot is shown in Figure 1c.

In order to perform metallographic and nano-indentation analysis, the welded sample was first cross-cut and then longitudinally cut to allow it to be mounted in an epoxy-resin. The sample was ground to ensure two flat and parallel opposite surfaces and then polished using various SiC papers with a P80 to a P2400 grit. The mirror-finishing step was initiated with a 9 μm diamond paste and then continued chemo-mechanically for 40 min in a colloidal silica suspension. Finally, the samples were rinsed in distilled water and ultrasonically cleaned for 10 min in ethanol. 

**Figure 2:**
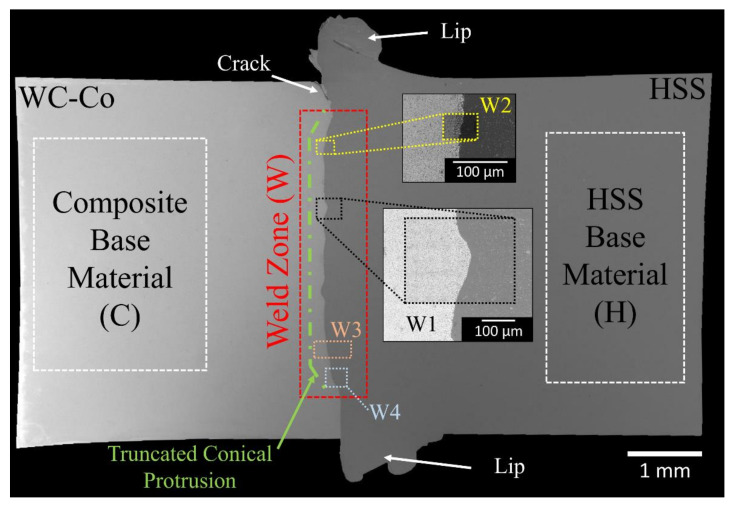
Field emission scanning electron microscopy (FESEM) image of the cross-sectional view of the high-speed steel/cemented carbide (HSS/WC-Co) welded joint after chemo-mechanical polishing. Notation: (C) composite base material, (H) HSS base material and (W) weld zone. Definition of subregions W1 to W4. The green dashed line indicates truncated conical protrusion. The white arrows indicate the extruded HSS lips and a crack in the upper part.

For ease of inspection, the sample was divided into three standard regions (Figure 2), namely, the weld zone (W), the composite base material (C) and the HSS base material (H).

### 3.1. HSS Base Material

Prior to optical microscopy (OM, DMI 3000M, Leica, Wetzlar, Germany), the surface of the HSS base material was etched by means of Murakami’s etchant for 20 s and gently dubbed for 20 s using a cotton dub drenched in a Nital agent. Field emission scanning electron microscopy (FESEM, JSM-7000F, Jeol, Tokyo, Japan) analysis was performed over a non-etched surface, both perpendicularly and along a 20°-tilt angle with respect to the electron beam, in secondary electron mode. Energy dispersive X-ray spectroscopy (EDS, Apollo SDD, EDAX, Foster City, CA, USA) maps were used to detect the distribution of the main alloying elements in selected regions. A more detailed microstructure analysis was performed with an atomic force microscopy (AFM, TI-950 Hysitron Triboindenter, Bruker Co., Billerica, MA, USA) in both force gradient and topographical modes. The same device was used for an inspection of the nanoscopic imprints.

The steel matrix was mechanically sensed [44] at 30 random sites by means of a nano-indentation test (TI-950 Hysitron Triboindenter, Bruker Co., Billerica, MA, USA) using a Berkovich indenter. A trial-and-error procedure was carried out before performing this test with the purpose of identifying the optimal indentation parameters that would prevent any indentation size effect. As a result, the following indentation parameters were determined: 10 mN peak load, 10 s holding time and 50 µN s^−l^ loading/unloading rate. Two nano-indentations (5 mN maximum load, 10 s holding time and 50 μm s^−1^ loading/unloading rate) were sufficient to discern the nature of the carbides embedded in the HSS matrix. By selecting a lower load for the carbides than for the HSS matrix, it was possible to sense different topological surface levels, and to protect the indenter from damage. A Poisson ratio value of 0.3 was used for the post-processing of the IC curves of the HSS matrix [45]. Indentation hardness and indentation modulus have been determined according to the actual ISO-14577 code [44], based on the Oliver and Pharr method [46].

**Figure 3:**
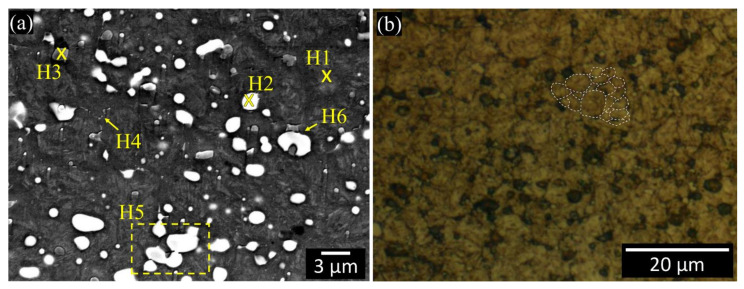
(**a**) FESEM microstructure of the cross-sectional view of the HSS base material after chemo-mechanical polishing. The crosses indicate pointwise EDS inspections; H1, H2, and H3 denote the martensite matrix, and the M_6_C and MC carbides, respectively; H4, H5 and H6 denote M_23_C_6_, similar M_6_C and dissimilar MC/M_6_C carbide clustering. (**b**) OM cross-sectional view of the HSS base microstructure after chemo-mechanical polishing and etching (Murakami’s etchant for 20 s and Nital agent for 20 s). The white dashed lines indicate the grain boundary structure.

**Figure 4:**
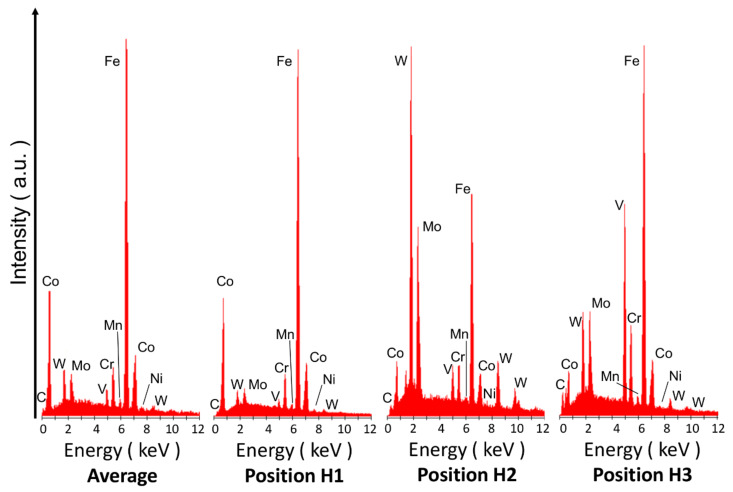
EDS area-averaged over the entire view-field and pointwise spectra at various positions (H1, H2, and H3) of the chemo-mechanically polished surface shown in Figure 3a.

The carbide particles were observed to be copiously present at a distance from the weld interface in the base material (Figure 3a). The martensitic structure is clearly discernible at location H1 (Figure 3a). This martensitic structure embeds two types of carbides, that is, M_6_C (H2) and MC (H3). Under secondary electron scattering (SEI) mode, the former carbides are shown to be bright, either round or elliptical in shape, and up to 4 µm in diameter, whereas the latter carbides are grey, polygonal and approximately up to 2 µm in size. The results of the pointwise chemical analysis, conducted by means of EDS, at regions H1, H2 and H3 in Figure 3a, are shown in Figure 4 and in Table 1 (at%). It was confirmed that the M_6_C and MC carbides contained relevant amounts of V, W, Mo and Cr elements. The M_6_C (H2) carbides are in fact much richer in W and Mo, while the MC (H3) carbides are much richer in V.

**Figure 5:**
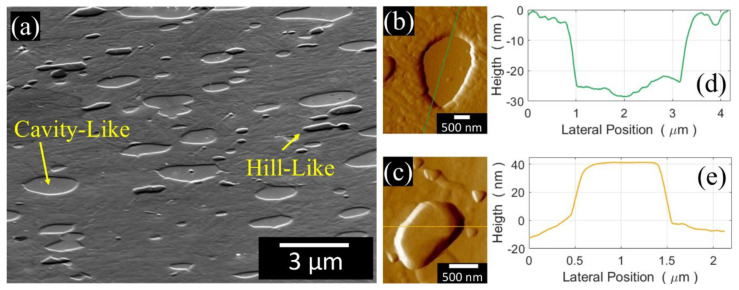
HSS base material after chemo-mechanical polishing. (**a**) Hill-like and cavity-like structures of MC and the M_6_C carbides in a cross-sectional view image obtained with a 20° tilted surface with respect to the FESEM electron beam; AFM force gradient in forward scanning mode of (**b**) an M_6_C cavity-like carbide and (**c**) MC hill-like carbides; topographical profiles of (**d**) an M_6_C cavity-like carbide and (**e**) MC hill-like carbides.

Furthermore, a third type of very tiny carbide (H4 in Figure 3a), denoted as M_23_C_6_, was observed. The M_6_C and MC carbides can be discerned by a gentle selective etching, using chemo-mechanical polishing with colloidal silica. Since the etching rate of these carbides differs from that of the matrix, three topological surface levels can be observed when the etched surface is tilted by 20° relative to the FESEM electron beam (Figure 5a). As a result, the M_6_C (resp. MC) carbides, which undergo a faster (resp. slower) etching rate than the martensitic matrix, appear as cavities (resp. hills) in Figure 5a. The results of the profilometry measurements of both carbides are compared in Figure 5b–e along with the results of the AFM scans. The H5 and H6 regions in Figure 3a indicate similar M_6_C and dissimilar M_6_C/MC carbide clustering, respectively. After selective etching and subsequent observation by means of OM, the average size of the martensite grains is 5–10 µm (Figure 3b).

**Figure 6:**
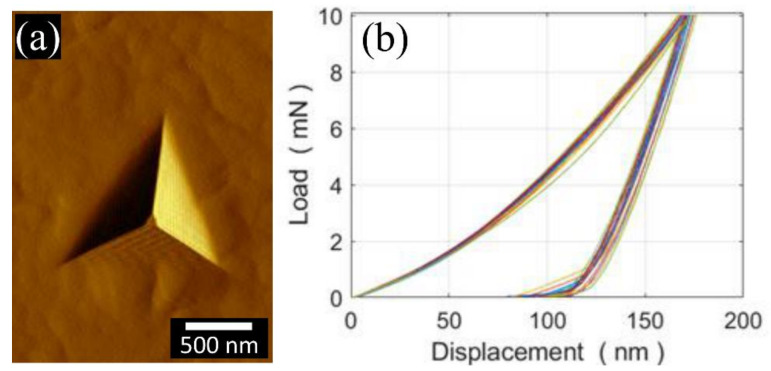
Nano-indentation across the HSS martensite matrix: (**a**) AFM force gradient in forward scanning mode of a typical imprint, (**b**) a selection of 20 (out of 30, for readability purposes) nano-indentation curves.

**Figure 7:**
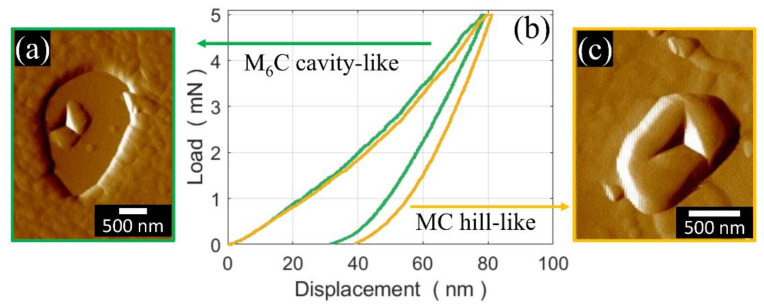
AFM force gradient in forward scanning mode of nano-indentation imprints over (**a**) an M_6_C cavity-like carbide and (**c**) an MC hill-like carbide, with (**b**) the relative nano-indentation curves.

The HSS matrix was sensed, by means of nano-indentation, at more than 30 random locations. A typical nano-indentation imprint is shown in Figure 6a. The indentation curves are shown in Figure 6b for only 20 (out of 30) indentations for clarity reasons. The measured indentation hardness and indentation modulus are 11.7 ± 0.4 GPa and 253 ± 6 GPa, respectively. The M_6_C and MC carbides were also sensed by means of nano-indentation, and the results of an AFM scan of the typical nano-indentation imprints and indentation curves are shown in Figure 7. As can be seen, the M_6_C and MC carbides have a similar indentation modulus (387 vs. 382 GPa) and indentation hardness (23 vs. 21 GPa).

### 3.2. Composite Base Material

**Figure 8:**
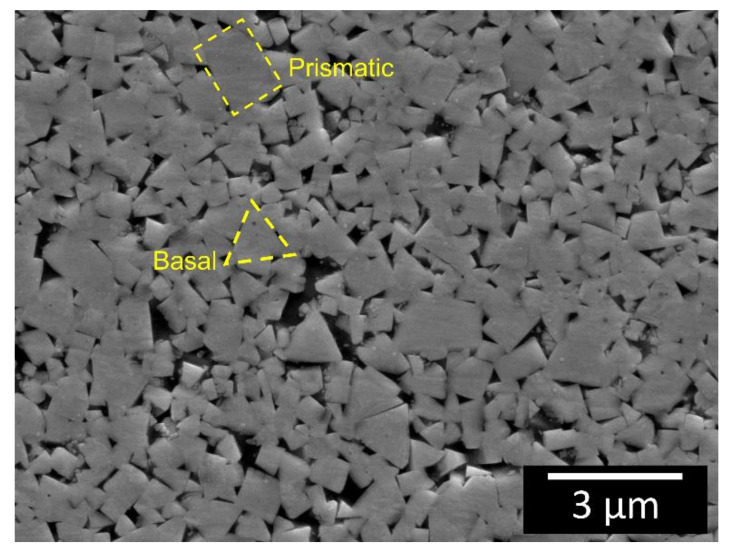
FESEM cross-sectional view of the composite base microstructure after chemo-mechanical polishing. The yellow dashed regions indicate prismatic- and basal-oriented WC particles.

The morphology of the WC-Co microstructure in the base material was analysed by means of FESEM. A total of 67 nIITs were required to account for the anisotropic mechanical properties of the WC particles [47]. It was not possible to inspect the Co matrix by means of nano-indentation due to its relatively low volume, compared to the WC phase. A Poisson ratio of 0.2 was assumed for the WC phase [48]. The microstructure of the WC-Co at a distance from the welding region is shown in Figure 8. The WC particles appear as faceted polygonal single crystals with either a triangular or rectangular shape. More detailed investigations, based on EBSD analysis, revealed that the particle size distribution of the WC particles was 400 ± 200 nm and that the triangular particles are essentially characterised by {0001} (basal) planes, while the rectangular particles are composed of {1010} planes [47]. The nano-hardness and indentation modulus of the basal-oriented crystals is 30 ± 2 GPa and 670 ± 43, respectively, compared with 23 ± 2 GPa and 576 ± 51, respectively, of the prismatic oriented crystals [47].

### 3.3. HSS/WC-Co Welded-Joint

The analysis carried out over the welding zone was consistent with that performed over the HSS and WC-Co base material regions. As the properties and characteristics of the welding zone, obtained by means of a CDW process, were very particular, we chose the weld zone (W) notation rather than the heat affected zone (HAZ) encountered in most common fusion welding processes. The sample surfaces that had to be indented were carefully prepared to fulfil the requirements [44] of flatness, smoothness and imprint separation. The CDW zone and the surrounding area were sampled, by means of nano-indentation, using two indentation arrays whose paths were perpendicular to the weld interface. Atomic force microscopy was used in the scanning mode to precisely locate the indentations and to observe the residual imprints. Each indentation was spaced approximately 5–15 μm apart, for both the HSS and the WC grains. The nano-indentations were basically aimed at assessing the indentation properties of the martensite matrix on the HSS side and the indentation properties of the WC grains in the composite side. However, it was very difficult to sense the indentation properties of the Co matrix, because of the small size of the Co domains between the WC grains. The nano-indentation imprints in the HSS were observed by means of dark-field optical microscopy.

The investigated joint sample revealed an optimal number of ejected molten HSS droplets, thus suggesting a good-quality welded joint, which was also confirmed from optical and FESEM observations. 

**Figure 9:**
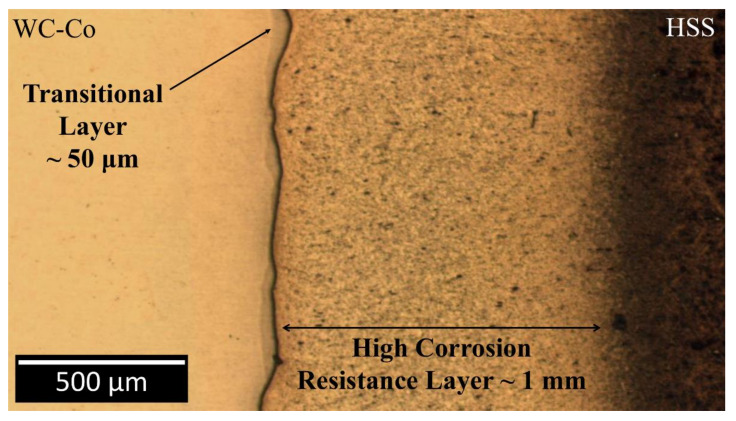
Optical image of the weld interface after chemo-mechanical polishing and chemical etching (Murakami 20 s and Nital 60 s). Evidence of an improved corrosion resistance layer of about 1 mm in thickness.

The FESEM image of the welded region is shown in Figure 2. The weld zone presents four regions, which are worth discussion and are denoted as W1 to W4. The central region of the weld interface contains the original conical protrusion of the HSS (Figure 2). However, it is reduced to a truncated cone, due to melting and its outward ejection, as a result of the current pulse discharge. The relatively low thermal conductivity of the HSS causes an appreciable softening of the steel at the weld region which, under the applied uniaxial pressure, is then extruded towards the periphery as a circular lip. A small crack is also evident in the lip region, at the interface, on the composite side. Optical observation (Figure 9) of the HSS side near the welding region after double etching (Murakami, 20 s and Nital, 60 s), revealed a bright (mildly etched) layer, up to 1 mm away from the welding line, and a dark (severely etched) region at a larger distance towards the bulk.

**Figure 10:**
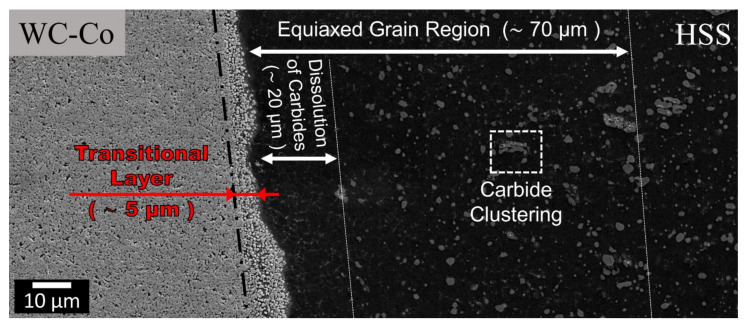
FESEM details of the W3 region in Figure 2. Evidence of a carbide dissolution region and an equiaxed grain region on the HSS side. Evidence of a transitional layer on the WC-Co side. The white rectangle indicates dissimilar HSS carbide clustering.

**Figure 11:**
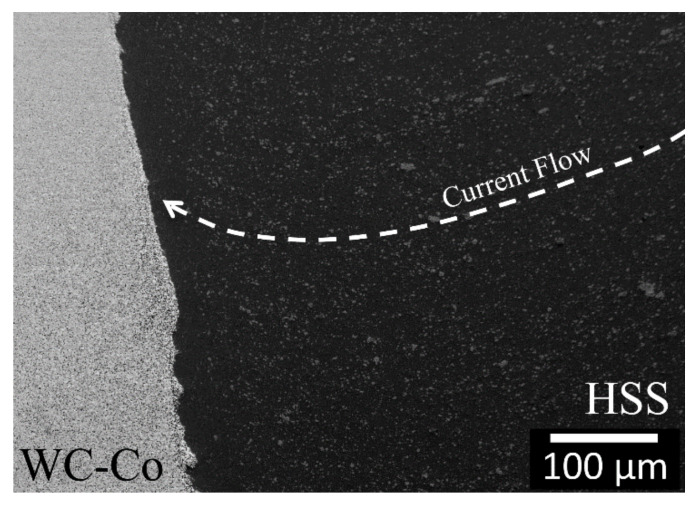
Higher FESEM magnification of the W4 region shown in Figure 2. The white dashed arrow indicates the co-alignment pattern of the dissolved carbides due to the current density flow.

**Figure 12:**
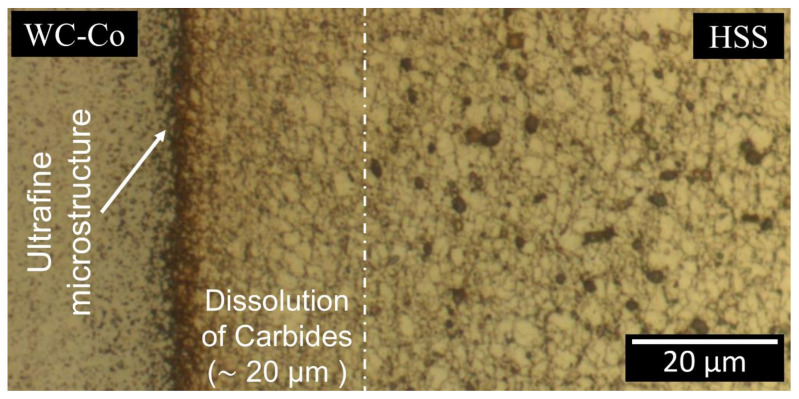
Optical image of the weld interface after chemo-mechanical polishing and chemical etching (Murakami’s 20 s and Nital 60 s).

**Figure 13:**
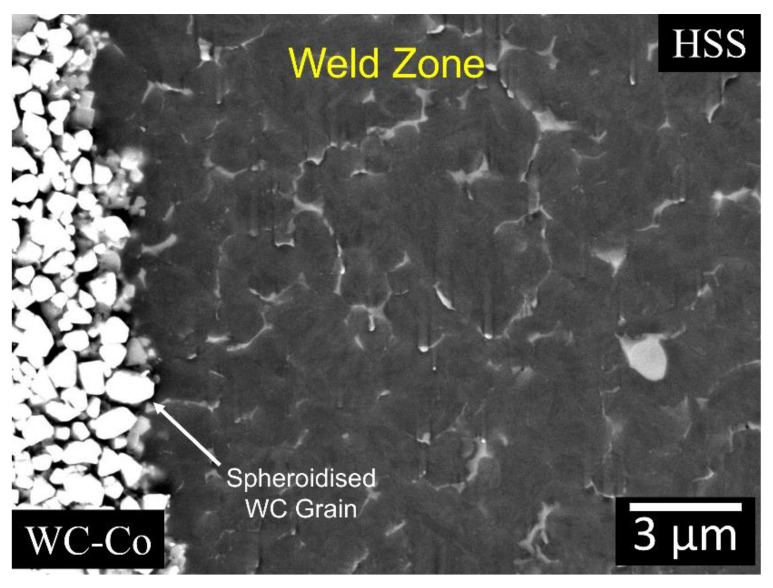
Higher FESEM magnification of the carbide dissolution region (Figure 10), details of the equiaxed grains on the HSS side and spheroidisation of WC particles on the composite side.

Figure 10 shows a thin carbide depleted layer (20 μm) on the HSS side, near the welding line (region W3), which is an indication of a significant dissolution of the initial carbides. The smaller carbides, originally present in a thicker region, covering a distance of 70 μm from the welding line (Figure 10, Figure 11 and Figure 12), have been dissolved in a martensitic matrix. Conversely, the larger carbides in the same region have basically remained unaltered. The martensitic matrix is composed of fine (of the order of 1 μm) equiaxed grains surrounded by grain boundary segregations, which appear as bright layers in Figure 13. 

The WC particles adjacent to the welding line, at the composite side, are more dispersed than those of the base material (Figure 10). This region is hereinafter denoted as “transitional layer”, taking inspiration from similar Ni/WC-Co welding joint studies [18,49]. This transitional layer is up to 50 μm thick and extends sideways along the welding line (Figure 9, Figure 10 and Figure 12). The composite region beyond the transitional layer is unaffected by the welding process (Figure 10).

By comparing the WC particles in the base material (Figure 8) with those in the transitional layer (Figure 13), it appears that the latter particles have been spheroidised by the welding process.

**Figure 14:**
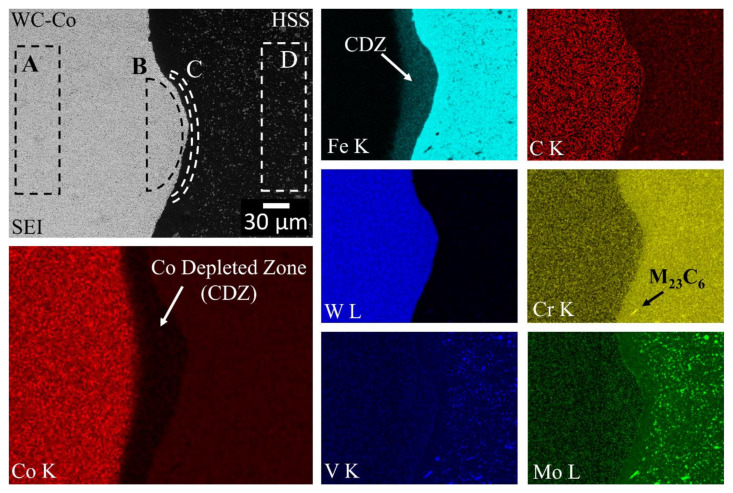
EDS chemical (at%) maps of the HSS/WC-Co interface (region W1 in Figure 2). Scan size of 355 × 281 μm. The dashed rectangles in the SEI image indicate the areas selected for a semi-quantitative EDS analysis, the results of which are listed in Table 2 and shown in Figure 16. The white arrow in the Co map indicates that the transitional layer corresponds to a Co depleted zone (CDZ), which is rich in Fe. The black arrow in the Cr map indicates an M_23_C_6_ Cr-based carbide.

**Figure 15:**
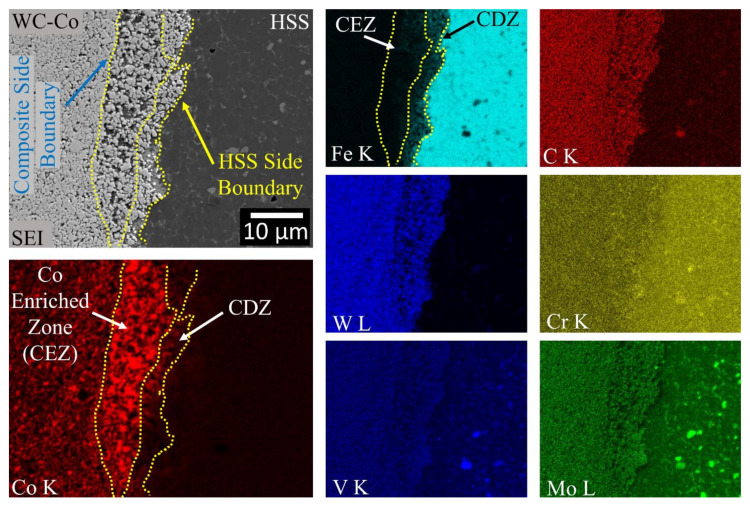
EDS chemical (at%) maps of the HSS/WC-Co interface (region W2 in Figure 2). Scan size of 50 × 40 μm. The white arrows in the Co map indicate that the transitional layer is composed of both a Co depleted zone (CDZ) and a Co enriched zone (CEZ). The blue and yellow arrows in the SEI image indicate composite and HSS side boundaries of the transitional layer, respectively.

**Figure 16:**
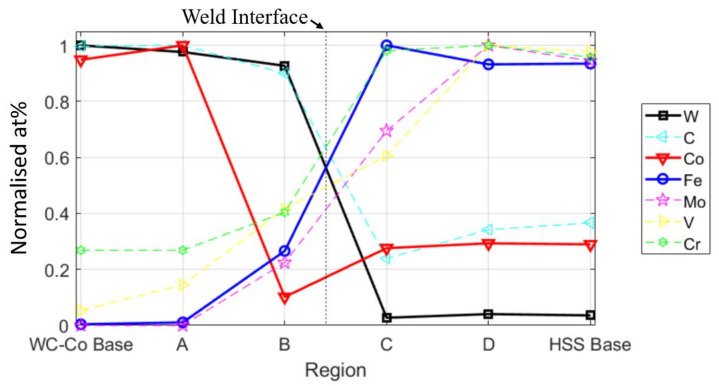
Concentration profiles of the main elements across the weld-line, based on the at% data listed in Table 2. The region selected for the EDS analyses is indicated in the SEI image of Figure 14.

The chemical changes induced by the welding process along the weld-line at two inspected regions (W1 and W2) were detected by means of EDS, and the results are shown in Figure 14 and Figure 15, respectively. Of the four regions at the interface, only region W1 was semi-quantitatively analysed by means of EDS (see the dashed regions in the SEI image in Figure 14). The results of the measurements in the W1 region are compared with both the composite and the HSS base material counterparts, numerically in Table 2 and graphically in Figure 16. It should be noted that the latter figure is expressed in terms of the normalised atomic percentage to separate the atom density effect of the contributing elements. The maps in both Figure 14 and Figure 15 indicate that most of C, W, Mo and V elements on the HSS side are combined as carbides. Thus, they are only present in a small number, if not completely absent, in the aforementioned carbide depleted layer on the HSS side. In Figure 14 and Figure 15, a few tens of micrometres thick layer in the composite side near the welding line is poor in Co and rich in Fe. The Co depleted zone (CDZ), next to the welding line, corresponds to the transitional layer shown in Figure 10 and Figure 12. Figure 15 suggests that there exists a Co enriched zone (CEZ), next the CDZ on the composite side, whose content is greater than the initial composite one.

**Figure 17:**
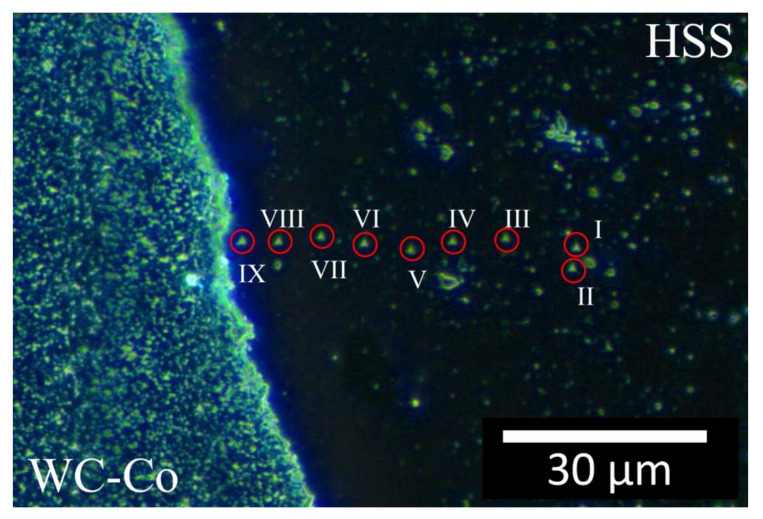
Dark Field Optical Microscopy image of an nIIT array across the weld-line. Here the transitional layer is very narrow, that is, less than 10 μm, as in Figure 10. The red circles and Roman numerals indicate the nine nano-indentation imprints on the HSS side; it should be pointed out that the profile continues on the WC-Co side, but it is not possible to discern the nano-indentation imprints by means of OM.

**Figure 18:**
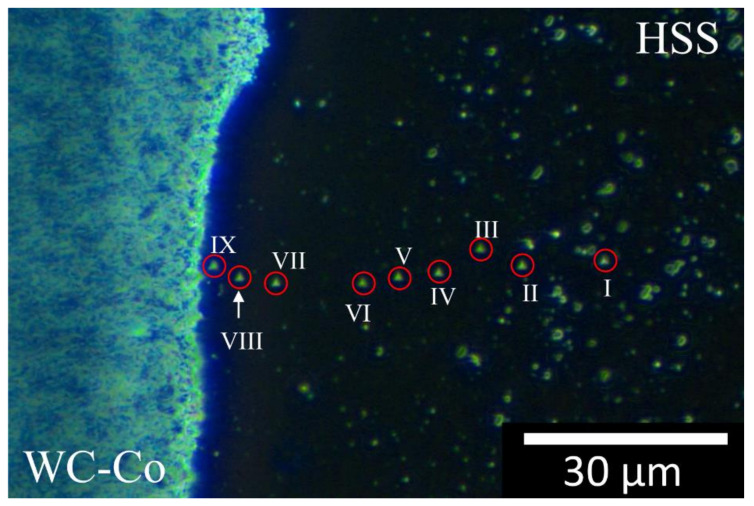
Dark Field Optical Microscopy image of an nIIT linear profile across the weld-line. Here the transitional layer is wide, that is, about 50 μm, as in Figure 9 (black arrow). The red circles and Roman numerals indicate nine (out of eleven) nano-indentation imprints on the HSS side; it should be pointed out that the profile continues on the WC-Co side, but it is not possible to discern the inherent nano-indentation imprints by means of OM.

**Figure 19:**
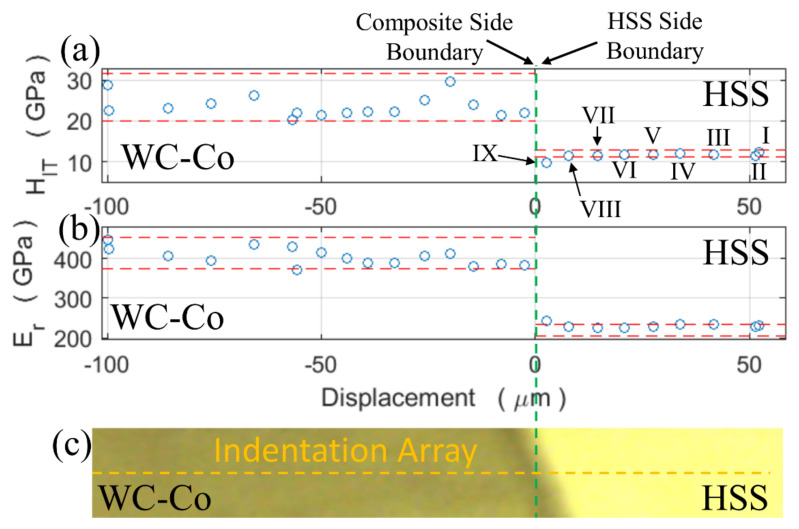
Indentation hardness (**a**) and reduced modulus (**b**) vs. displacement along the nIIT array across a narrow (less than 10 μm) transitional layer. The red dashed lines show the ranges of indentation hardness and reduced modulus characteristics of the base materials, (**c**) is the optical microscopy image taken from the positioning system of the nano-indenter machine. The Roman numerals indicate the nine nano-indentation imprints on the HSS side, according to Figure 17. The green dashed line indicates the HSS/WC-Co interface. The green dashed lines indicate the composite and the HSS side boundaries of the transitional layer. The thickness of the transitional layer is less than the distance between two nano-indentations, thus both its boundaries lie on the same dashed line.

**Figure 20:**
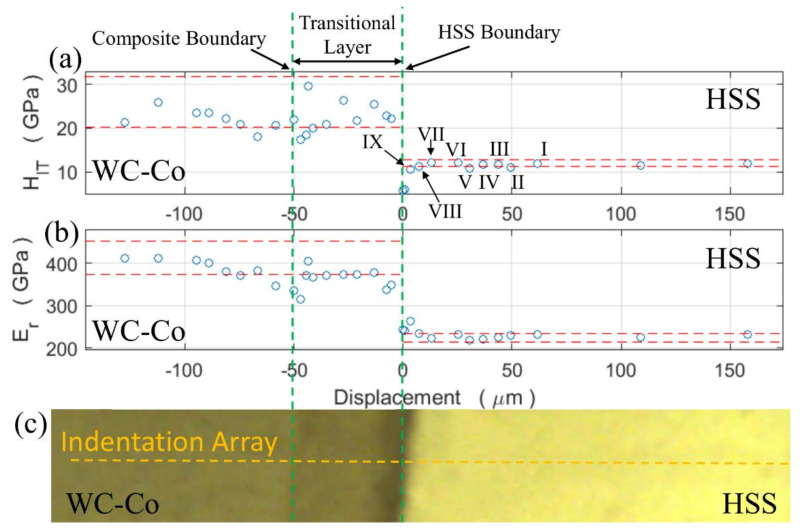
Indentation hardness (**a**) and reduced modulus (**b**) vs. displacement along the nIIT array across a wide (about 50 μm) transitional layer. The red dashed lines show the ranges of indentation hardness and reduced modulus characteristics of the base materials, (**c**) is the optical microscopy image taken from the positioning system of the nano-indenter machine. The Roman numerals indicate nine nano-indentation imprints on the HSS side, according to Figure 18. The green dashed lines indicate the composite and the HSS side boundaries of the transitional layer.

Figure 17 and Figure 18 are optical dark field images that show the two parallel nano-indentation arrays aligned perpendicularly to the welded line. The nano-indentations on the HSS side are visible (see the Roman numerals), while those on the composite side are barely visible. Note that, the spacing between indentations I and II in Figure 17 was accidentally smaller than the minimum value specified by the International code [44]. Nevertheless, the indentation properties given by these two indentations (Figure 19) compared well with those of other indentations and therefore their values contributed to the present analysis. The measured indentation hardness and indentation modulus profiles are shown in Figure 19 and Figure 20 and compared with the displacement across the welding line. The horizontal red dashed lines across the three sample regions (C, H and W) determine the (95 %) uncertainty bands of *H_IT_* and *E_r_* about their mean values, although the situation is complicated by the orientation of the WC crystals for the composite as described in Section 3.2.

The two indentation arrays cross the transitional layer at layer thicknesses of 5 a 50 μm, values which represent two quite different situations in terms of WC particle separation. It should be noted that, while approximately the same statistics are obtained for the indentation hardness of the WC crystals in both the transitional layer and the base material, the indentation modulus, due to its innate sensitivity to the surrounding Co matrix, enables the extent of the transitional layer to be discerned more clearly and, thus, that of its boundaries.

## 4. Discussion

The microstructure of the HSS base material (Figure 3a and Figure 5a) includes the martensite matrix and various carbides of different stoichiometry. The nature of these carbides is similar to that found in other works for the same M35 HSS [40,42,50,51,52] and more generally for other HSS materials (e.g., [43,53]). According to reference [40], the carbide population found here is mainly composed of MC carbides that are very rich in V (VC), and M_6_C carbides very rich in W and Mo (Fe_3_W_3_C). Skakov et al. [43] suggested that Mo can replace W, while V can replace Fe in M_6_C carbides. It is believed that the elongated and submicrometric size carbides observed in Figure 3a are of the M_23_C_6_-type [40]; however, a detailed inspection, by means of EDS, was difficult owing to their very small size.

The MC and M_6_C carbides exhibited a similar nano-indentation hardness (21 vs. 23 GPa) and indentation modulus (387 vs. 382 GPa). This is in contrast with previous observations [54], in which MC carbides displayed a greater nano-indentation hardness and higher reduced modulus than M_6_C carbides. The reason for such an inconsistency is twofold. First, the present hardness analysis is based on only two nano-indentations, a number which is insufficient to represent the correct statistics of the actual properties of the carbide. Second, the present carbides are slightly smaller than those investigated in the previous research [54], and the steel matrix embedding the carbides may affect the nano-indentation measurements. Nonetheless, the carbides were found to be considerably harder and stiffer than the steel matrix.

In the present case, the HSS matrix is martensite. As the martensite finish temperature for these steels is frequently below room temperature, the co-existence of some retained austenite cannot be excluded. The EDS analysis indicated that the martensite was rich in Co (Table 1). 

As per the indentation properties of the HSS, we selected a maximum force of 10 mN, as in reference [55], and found a reduced modulus of 224 GPa (253 GPa indentation modulus and 173 ± 3 nm penetration depth), which compares well with the 221 GPa found for a similar HSS steel. This penetration depth is similar to the 200 nm used by other authors [56], who found a reduced modulus of ~230 GPa for another HSS and, moreover, is quite close to the Young modulus (210 GPa) of a typical steel [56]. Our indentation modulus (253 GPa) is also similar to that of martensite (~250 GPa) [57]. The measured nano-hardness of the martensite, that is, 11.7 GPa, compares well with the ~13 GPa at a maximum depth of 200 nm [56], but it is slightly larger than the 9.41 GPa of an as-cast HSS [55].

The investigation of the microstructure and indentation properties of the WC-Co composite analysed in this work are detailed in a companion paper [47].

Capacitor discharge welding has a unique potential for the production of efficient, net-shaped and sound joints for either similar metals and alloys or dissimilar alloys and ceramics [33].

During the early stage of the CDW process, the very high current density is forced to pass through the HSS tip, which causes the tip to melt fast. Evidence of the high current density flow effect can be seen in Figure 11, that is, the co-alignment pattern of the dissolved carbides (dashed arrow) near the weld interface as a result of the strong local thermal non-equilibrium condition. The high electric field perpendicular to the weld-line induced a radial magnetic field, which forced most of the liquid metal formed at the weld zone to be radially expelled outwards from the contact region as flash [33]. As WC has a higher thermal conductivity than HSS, the HSS next to the HSS/WC-Co interface was rapidly quenched (~10^3^ °C/s [33]). As heating was confined to the HSS/WC-Co contact interface, the steel matrix transformed into a very fine microstructure, while the steel carbides underwent a severe dissolution. Both chemical and microstructure gradients can be expected when considering the HSS base material.

The initial conical projection in the HSS appears as a truncated conical protrusion at the central region of the welded joint, as a result of the tip lost as ejected molten droplets (Figure 2). The remaining hot protrusion of the HSS underwent a plastic flow towards a composite surface, which was driven by the imposed uniaxial pressure. The combination of surface melting and plastic flow of the HSS determined an uneven microscale weld interface (Figure 2), although it can macroscopically be considered flat. It was reported that an uneven weld interface exhibited an extended contact area, thereby enhancing the strength of the CDW joint [58].

A small crack was observed at the periphery of the weld zone on the composite side, near the extruded lip. This crack originated from and propagated upon rapid cooling as a consequence of the build-up of large thermal stresses, due to the large differences in thermal contraction between the two dissimilar joining materials and of the residual stresses due to phase transformations on the HSS side. Interestingly, the crack did not propagate during the preparation of the welded sample after cutting or after polishing, although we expected the presence of non-negligible residual stresses. This is a useful indication of an appreciable intrinsic toughness of the produced WC-Co/HSS CDW joint.

No porosity was detected at the welded region. This may be attributed to an optimal selection of the electric energy density and the imposed load.

The carbide-free layer (20 μm thick) on the HSS side is composed of a very fine (~1 μm) equiaxed grain martensitic matrix (Figure 10 and Figure 13). It developed along most of the weld-line of the truncated protrusion. This fine equiaxed martensitic structure extended up to a distance of 70 μm from the weld-line (Figure 10). The combination of a homogeneous and refined grain structure matrix in the weld zone regions is an outstanding feature of CDW. This is determined by the very high temperature achieved in a very short time during heating and cooling (of the order of 10^3^ °C/s [33]) and by the ejection of any liquid metal produced during an electric discharge. The consequent advantages are: (a) most of the liquid metal that forms upon heating is eliminated, (b) the interface temperature is decreased and (c) the contacting regions are homogenised (as a result of the high temperature carbide dissolution in the solid state).

However, certain equiaxed grain boundaries may retain some segregations rich in Cr and Mo (Figure 15), when these regions are exposed to a high temperature near the melting point of the matrix. Such a characteristic equiaxed martensitic microstructure, with carbide segregation at the grain boundaries, is similar to that observed in AISI HSS M35 after casting (see Figure 1a in reference [40]), except for the microstructure resolution. The interface microstructure of HSS after CDW is much finer than that obtained after casting. Such a fine microstructure across the welding line can readily be appreciated by means of optical microscopy after HSS etching (Figure 12 and Figure 13).

Furthermore, the presence of a high approximately 1 mm thick corrosion resistant layer, can also be observed in Figure 9 after Nital etching of the HSS side near the welding line. Although 20 s is normally sufficient for the base material to be etched, 60 s were in fact required to etch such a thin interface layer. This particular behaviour is associated with a partial dissolution of Cr-carbides which, in turn, leads to a possible beneficial increase in the Cr content of the martensitic matrix near the interface. While carbide dissolution was proved, by means of FESEM, across a 70 μm-thick layer from the welding line, the actual enrichment of Cr in the matrix could not be confirmed, due to the difficulty of observing the tiny Cr-carbides, even with FESEM. This specific aspect requires further research. 

The WC-Co composite facing onto HSS at the interface is affected by the massive plastic flow of the HSS in two ways: (a) the WC particles spread, and their relative separation therefore increases, (b) and the liquid Fe infiltrates (replacing Co) among the WC particles. Both phenomena promote the formation of the so-called transitional layer. Previous welding studies suggested that the transitional layer emerged as a result of the presence of tiny WC grains in very fine WC-Co composites, but was absent in coarse WC-Co composites [18,30,49]. Moreover, it was depressed by the presence of internal stresses at the interface [18,59]. 

A further impact of CDW on the composite WC particles is their spheroidisation at the weld interface (Figure 13). 

Figure 14 and Figure 15 show the EDS maps of the weld region, near the HSS side. As can be observed, C, Mo, W and V are mainly linked to the carbides, while Cr and Co are preferentially in a solid solution in martensite, thereby supporting the pointwise EDS results pertaining to the HSS base material shown in Figure 3a and Table 1. The morphology of the Cr-carbide (black arrow) in Figure 14 resembles that of a M_23_C_6_ carbide contained in a similar (AISI M35) steel [40].

Figure 16 shows semi-quantitative area-averaged profiles of the alloying elements detected by means of EDS over the welding line. The weld interface is located between the B and C regions, which span a distance of 50 μm. The base material of the composite is represented by the maximum normalised contents of Co, W and C, while Cr and V are present, albeit in minimal contents, in the matrix with Co [60]. On the other side, HSS shows the maximum normalised contents of Fe, Mo, V, Cr, whereas Co, C and W are observed in minimum. The main migrating elements are Co, from the composite side, and Fe from the opposite HSS side. It should be noted that all the analysed elements were associated with nearly-melting regions and that their actual migration was more likely of a convection type (plastic flow) than of a pure diffusion type. Fe migrates towards the composite side, along with other elements, such as Mo, V, and Cr, as their contents at B are much larger than those available in the original composite base material. Conversely, Co migrates backwards, as it is pushed back by the Fe (and Mo, V and Cr) matrix. The Co content at B is much lower than that originally present in the composite base material, while it remains unaltered on the HSS side (C, D and the HSS base material). The Co content in A is in fact slightly larger than that of the original composite base material. The Co map in Figure 15 confirms the presence of a Co enriched zone at a local protrusion of the weld zone, which should be compared with its original content in the composite base material (leftmost) and that in the adjacent Co depleted zone (rightmost). Accordingly, a remarkable infiltration of Fe can be observed in the Co depleted region (Figure 14 and Figure 15), while only traces of Fe are detected in the Co enriched zone (Figure 15).

As the Mo, V, W and C on the HSS side are mainly linked to carbides, rather than to a solid solution with the Fe matrix, and since the C region represents the carbide-free layer (Figure 10 and Figure 14), the Mo, V, W and C contents are considerably lower (30–40% less) in the C region than in the initial HSS base material (Table 2 and Figure 16). On the other hand, the Fe content in the C region is slightly increased.

It should be noticed that, in both the B and C regions, the carbon content decreased with respect to that of both base materials. Moreover, no other regions showed larger carbon contents than that of the base material. These results excluded any diffusion of carbon, despite the presence of other elements which may be diffused to some extent. A considerable amount of carbon may have evaporated during the CDW process. It has frequently been reported that some ternary mixed carbides (e.g., M_6_C and M_12_C) play a detrimental embrittling role at the WC/Steel weld zone. The growth of such phases is controlled by the diffusion of C from WC to steel [21]. The present EDS results exclude any carbon diffusion from the WC-Co to the HSS. Moreover, the dissolution of carbides, induced by high intensity currents, prevents the formation of brittle phases.

The nano-instrumented indentation array surveys (Figure 19 and Figure 20) carried out on the welding line surprisingly show that the indentation properties of the equiaxed-grain martensite at the HSS boundary exhibit comparable values to those of the HSS base material, although the grain size of the welded region is much finer than that of the HSS base material. These unexpected identical properties can be explained considering two opposing contributions. The larger hardness of the finer CDW martensite is counterbalanced by the softening due to the local dissolution of carbides, which are retained in the matrix of the original coarser martensite structure. The nano-indentation array across a thinner transitional layer than 10 μm in Figure 19 shows that the indentation hardness is slightly lower in the transitional region and that the reduced modulus is slightly larger than that of the martensite in the HSS base material. Conversely, the measured indentation hardness drops significantly over the wider (50 μm) transitional layer near the welding interface (Figure 20). The reduced modulus increases at the HSS side boundary of the transitional layer, from the HSS reduced modulus value to that of the composite base material. The EDS results across the transitional layer attest that Co is replaced by Fe, although the indentation properties of the final WC-(Co,Fe) composite do not change significantly in comparison to that of the original composite base material. The indentation properties suddenly fall off, at the composite side boundary (Figure 20) across the transitional layer, especially in the reduced modulus. Such a fall can in general be explained by considering the increased dispersion of the WC particles. As shown in Figure 15, the separation between the WC particles is larger at the composite-side boundary than in the other regions of the transitional layer. In fact, whenever the WC particles embedded in a larger Co matrix, relative to the original composite, which are found at the composite boundary, are indented, a similar hardness of the WC particles can be expected before and after welding, but also a decrease in the reduced modulus of the WC grain after welding, due to a larger softer matrix [61]. Thus, the collected results suggest that the CDW zone is tougher than a typical heat affected zone of a conventional fusion welding process, due to the presence of an extremely fine martensitic-carbide free microstructure. However, a slight softening is detected in a narrow region at the composite side boundary, due to the presence of a larger softer matrix which embeds the WC grains.

Thus, the welded regions resulting from CDW and other conventional fusion welding technologies differ in several ways. At a microstructural level, the welding zone of the CDW is much smaller than the typical HAZ of fusion welding processes. Moreover, the grain structure is equiaxed, relatively fine and homogeneous. The thickness of the welded region here extends to about 70 μm on the HSS side and to 50 μm on the composited side. However, the high local temperature and high electric energy density at the welding interface cause a dramatic dissolution of very fine carbides. These particles are too small to be detected by means of FESEM. This dissolution may span over a thickness of 1 mm on the HSS side. Surprisingly, it was found that this layer exhibited enhanced corrosion resistance, which may be considered an added value of the HSS-side welded zone. An inspection of the nano-indentation highlighted that the martensite matrix of the HSS exhibited comparable indentation properties to the HSS base material, except for a few micrometre- thick layers adjacent to the transitional layer on the HSS side. The indentation properties of the WC particles in the transitional layer on the composite side had comparable indentation properties to those dispersed in the base material. However, a slight softening was evidenced on both the HSS-side and at the composite-side boundaries at the transitional layer. From a chemical viewpoint, it was observed that alloying elements migrated over a range of up to 60 μm from the weld interface on both the HSS and composite sides.

## 5. Conclusions

A sound dissimilar solid-state WC-Co/HSS butt joint was successfully fabricated using a novel capacitor discharge welding process, with a conical projection in the HSS rod. A correlation was determined between the chemical composition (EDS), nano-indentation properties and microstructure (by means of optical, atomic force and scanning electron microscopy). The residual hot HSS truncated cone favoured a plastic flow of the HSS which, in turn, uniformly wetted the WC-Co surface. The bonding between the two dissimilar surfaces occurred both chemically and structurally, via the formation of an intermediate transitional layer with two boundaries for the HSS and WC-Co base materials, respectively. The indentation properties at the HSS boundary are comparable with those of the HSS bulk. Selective etching evidenced the presence of a high corrosion resistance layer (1 mm thick) on the HSS side. Conversely, the indentation properties of the composite boundary decreased appreciably, especially in terms of reduced modulus, as a result of an increased dispersion of WC particles in the surrounding soft matrix. Structurally, the martensite matrix adjacent to the HSS boundary has been found to be particularly fine and carbide free. Both features indicate an innate toughness of the CDW joint, although high thermal and residual stresses build-up on cooling as a result of the large differential thermal expansion of the joint as well as the phase transformations in the steel and Co matrices. The relatively short heating time does not permit any significant atomic diffusion, except for a very thin layer near the WC-Co boundary. Here, a significant amount of Fe, which was originally dissolved in the HSS base material, migrated towards the composite (perpendicularly to the welding interface), as it was associated with the HSS plastic flow, and ultimately infiltrated among the WC particles, thereby replacing the Co binder. The latter, in turn, migrated backwards to the composite region, while a counterflow of WC particles was directed towards the welding interface. The increased separation between the WC grains, in favour of a larger amount of soft matrix, indicated an increased toughness of the joint at the composite boundary, although the involved layer thickness was not found to be uniform along the welding line. However, any evidence of alloying element migration over large distances should be attributed to the convection flow during the electric discharge rather than to atomic diffusion. 

## Figures and Tables

**Table 1:** Semi-quantitative pointwise EDS analyses of various positions indicated in Figure 3a, compared with the average composition of the complete view-field. The results refer to the spectra in Figure 4; each position was repeated three times and the inherent results are listed here in terms of average atomic percentage.

Element	at %			
	Average	H1	H2	H3
**C**	6.99	5.34	10.33	21.43
**Mo**	2.57	1.65	16.97	7.33
**V**	2.16	1.21	5.04	28.61
**Cr**	4.53	4.53	5.38	5.32
**Mn**	0.94	0.47	0.49	0.47
**Fe**	74.55	79.7	42.42	30.05
**Co**	5.58	5.58	3.58	2.11
**Ni**	0.55	0.33	0.30	0.54
**W**	2.12	1.18	15.50	4.14

**Table 2:** Semi-quantitative EDS analyses of various regions indicated in Figure 14 and Figure 16, compared with the EDS average composition of both the high-speed steel (HSS) base and composite base materials. The tungsten carbide (WC) base material composition data are taken from [47].

	at %					
	WC Base	A	B	C	D	HSS Base
**C**	19.04	19.09	17.18	4.56	6.53	6.99
**Mo**	0	0	0.61	1.89	2.72	2.57
**V**	0.12	0.32	0.92	1.34	2.21	2.16
**Cr**	1.27	1.27	1.91	4.64	4.73	4.53
**Mn**	0.35	0.35	0.45	0.42	0.93	0.94
**Fe**	0.32	0.85	21.19	79.74	74.33	74.55
**Co**	18.29	19.28	1.96	5.32	5.65	5.58
**Ni**	0.67	0.88	0.75	0.47	0.51	0.55
**W**	59.36	57.95	55.03	1.62	2.40	2.12
